# Peste des Petits Ruminants Virus-Like Particles Induce a Potent Humoral and Cellular Immune Response in Goats

**DOI:** 10.3390/v11100918

**Published:** 2019-10-05

**Authors:** Feihu Yan, Logan Banadyga, Yongkun Zhao, Ziqi Zhao, Zachary Schiffman, Pei Huang, Entao Li, Cuiling Wang, Yuwei Gao, Na Feng, Tiecheng Wang, Hualei Wang, Xianzhu Xia, Chengyu Wang, Songtao Yang, Xiangguo Qiu

**Affiliations:** 1 Key Laboratory of Jilin Province for Zoonosis Prevention and Control, Changchun Veterinary Research Institute, Chinese Academy of Agricultural Sciences, Changchun 130122, Jilin, China; yanfh1990@gmail.com (F.Y.); zhaoyongkun1976@126.com (Y.Z.); gaoyuwei@gmail.com (Y.G.); fengna0308@126.com (N.F.); wgcha@163.com (T.W.); xiaxzh@cae.cn (X.X.); 2 Special Pathogens Program, National Microbiology Laboratory, Public Health Agency of Canada, Winnipeg R3E 3R2, Manitoba, Canada; logan.banadyga@canada.ca; 3 Department of Medical Microbiology, University of Manitoba, Winnipeg, Manitoba, Canada; Zachary.Schiffman@canada.ca; 4 College of Veterinary Medicine, Jilin Agricultural University, Changchun 130122, Jilin, China; zhaoziqi1989@yeah.net (Z.Z.); hp19932015@163.com (P.H.); 5 College of Veterinary Medicine, Huanan Agricultural University, Guangzhou 510642, Guangdong, China; liet1990@sina.com; 6 Xinxiang medical university, Xinxiang 453003, Henan, China; wangcl0229@163.com; 7 College of Veterinary Medicine, Jilin University, Changchun 130122, Jilin, China; whl831125@163.com

**Keywords:** peste des petits ruminants virus, baculovirus, virus-like particles, humoral response, cellular response

## Abstract

Peste des petits ruminants is a highly contagious acute or subacute disease of small ruminants caused by the peste des petits ruminants virus (PPRV), and it is responsible for significant economic losses in animal husbandry. Vaccination represents the most effective means of controlling this disease, with virus-like particle (VLP) vaccines offering promising vaccine candidates. In this study, a PPRV VLP-based vaccine was developed using a baculovirus expression system, allowing for the simultaneous expression of the PPRV matrix (M), hemagglutinin (H), fusion (F) and nucleocapsid (N) proteins in insect cells. Immunization of mice and goats with PPRV VLPs elicited a robust neutralization response and a potent cellular immune response. Mouse studies demonstrated that VLPs induced a more robust IFN-γ response in CD4^+^ and CD8^+^ T cells than PPRV Nigeria 75/1 and recruited and/or activated more B cells and dendritic cells in inguinal lymph nodes. In addition, PPRV VLPs induced a strong Th1 class response in mice, as indicated by a high IgG2a to IgG1 ratio. Goat studies demonstrated that PPRV VLPs can induce the production of antibodies specific for F and H proteins and can also stimulate the production of virus neutralizing antibodies to the same magnitude as the PPRV Nigeria 75/1 vaccine. Higher amounts of IFN-γ in VLP-immunized animal serum suggested that VLPs also elicited a cellular immune response in goats. These results demonstrated that VLPs elicit a potent immune response against PPRV infection in small ruminants, making PPRV VLPs a potential candidate for PPRV vaccine development.

## 1. Introduction

Peste des petits ruminants virus (PPRV) is the causative agent of peste des petits ruminants (PPR), a highly contagious disease that affects small ruminants and results in extremely high rates of mortality and morbidity. PPR is currently endemic in most of Africa, South Asia, the Middle East, and China [1]. PPRV is a member of the genus *Morbillivirus*, family *Paramyxoviridae* [2], and has been divided into four lineages based on the genes encoding the capsid and fusion proteins [3,4,5]. Mature virus particles are spherical or elliptical in shape and range in size from 150 nm to 700 nm in diameter [6]. PPRV consists of a 15,948 bp single-stranded, non-segmented negative-sense RNA genome that encodes a total of six structural proteins and two non-structural proteins [7,8]. Among the structural proteins, the M protein contributes to the formation of virus particles and is also a driving force in virus budding [9]. In addition, the F and H proteins are the most immunologically relevant determinants of protection against morbilliviruses, containing several T cell epitopes and the majority of neutralizing antibody epitopes [10,11,12]. Both F and H induce immune protection in the host, but the H protein stimulates a stronger humoral immune response and leads to the production of more neutralizing antibodies than the F protein [10,13,14]. The N protein, which is the most abundant PPRV protein, is not only a major structural protein but can also induce a cytotoxic T-lymphocyte (CTL) response [15,16].

PPR is classified as a notifiable disease by the World Organization for Animal Health [1]. According to the United Nations Food and Agriculture Organization, it is estimated that PPR resulted in economic losses of USD$2.9 million/year during 2012–2017 due to morbidity, mortality, production losses and treatment costs in member nations of the South Asian Association for Regional Cooperation (SAARC) [1,17]. The disease is characterized by fever, mouth sores, diarrhea and pneumonia and is capable of causing death in both sheep and goats [18]. In goats, mortality can be as high as 100%, and in some cases, even peracute PPRV infection can cause death. The first PPR outbreak in China was reported in Tibet in 2007 and was effectively contained through slaughter, vaccination and restrictions in animal movement in PPR-infected areas [19]. PPR later re-emerged in Xinjiang, China, in December 2013 and rapidly spread to most parts of the country in 2014, after which, it was substantially controlled nationwide [20]. In 2015, a national eradication program for PPR was implemented in China to counter the serious threat that PPR poses to animal health and safety, and, as a result, PPRV is expected to be eradicated nationwide by 2020 [2]. Thus, it is particularly important to prevent PPR recurrence, ideally through the vaccination of animals with a robust and effective vaccine.

Vaccination is the most effective method for preventing recurrence of PPRV infection. Attenuated PPRV Nigeria 75/1 and Sungri 96 are among the most widely used vaccines worldwide and are considered to be completely attenuated, safe, and potent. These two vaccines also offer lifelong protection to the immunized animals [21]. However, these live-attenuated vaccines have poor thermal stability and are, therefore, difficult to maintain in a cold chain, especially in subtropical areas. Live-attenuated vaccines also run the risk of undergoing reversion to a virulent phenotype, although this phenomenon is extremely rare and has not been seen for either the PPRV Nigeria 75/1 or the Sungri 96 live attenuated vaccines in the over forty years they have been used [22]. Importantly, another drawback of the use of these vaccines is the inability to differentiate vaccinated animals from infected animals, which complicates control and regulatory measures [23]. VLPs containing multiple structural proteins are structurally similar to native PPRV virus particles [24]. Because of their strong antigenicity, VLPs can be easily recognized by antigen-presenting cells and B cells, and are capable of inducing strong humoral and cell-mediated immune responses [25]. 

Although previous studies have been successful in producing PPRV VLPs using sequences without codon optimization, the yield of VLPs is often low (3–4.5 mg/L) [26], thus increasing production costs. In addition, previous studies have demonstrated that VLPs made up of only the PPRV M and N proteins cannot induce the production of neutralizing antibodies in mice [27]. To our knowledge, there is currently no report of the production of PPRV VLPs from the simultaneous expression of the four structural proteins (M, F, H and N). In this study, VLPs produced from the expression of codon-optimized M, F, H, and N were developed using a baculovirus system for protein expression in insect cells. The VLPs were used to immunize mice and goats, and the results demonstrate that they induced both humoral and cell-mediated immune responses.

## 2. Materials and Methods

### 2.1. Cell Culture and Viruses

*Spodoptera frµgiperda* Sf9 (ATCC® CRL-1711^TM^) insect ovary cells in suspension culture were grown in Sf-900^TM^ II serum-free medium (Life Technologies, USA) at 27 °C on a temperate orbital shaker at 200 rpm. Vero (ATCC^®^ CCL-81^TM^) cells were grown in Dulbecco’s modified Eagle medium (DMEM, Thermo Fisher, USA) supplemented with 10% fetal bovine serum (FBS, Gibco, Thermo Fisher, USA) at 37 °C with 5% CO_2_ for PPRV propagation and titration. Nigeria 75/1, the vaccine strain of PPRV, was purchased from Tecon, Xinjiang, China.

### 2.2. Construction of Bacmid Transfer Plasmid

The open reading frames (ORFs) for the PPRV (China/Tibet/geg/07-30) M, F, H and N genes were obtained from NCBI (Gen-Bank Access NO.FJ90530401). Codons in the four ORFs were optimized based on Sf9 cells to eliminate rare codons (Sangon Biotech, China).

**Figure 1:**
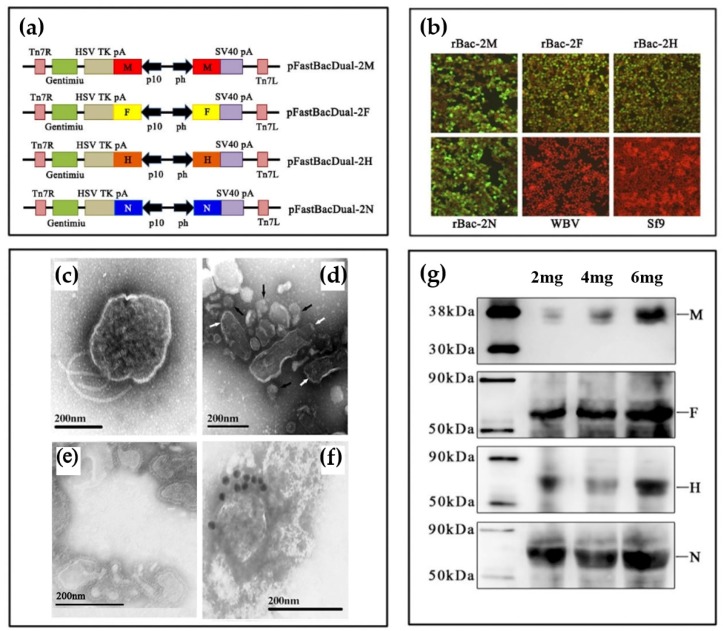
Generation and characterization of peste des petits ruminants virus (PPRV) virus-like particles (VLPs). (**a**) Schematic diagrams are presented for the recombinant plasmids pFastBacDual-2M, pFastBacDual-2F, pFastBacDual-2H, pFastBacDual-2N. (**b**) Detection of the expression of M, F, H and N. Sf9 cells were mock-infected or infected with recombinant baculoviruses rpFBD-2M, rpFBD-2F, rpFBD-2H, rpFBD-2N or wild-type baculovirus (WBV). Expression was evaluated by IFA using a mouse anti-PPRV M polyclonal antibody, mouse anti-PPRV F polyclonal antibody or sheep polyclonal antibodies against N and F. (**c–f**) Transmission electron microscopy images of virus and VLPs preparations. Native peste des petits ruminants virus (PPRV) (c), residual baculoviruses (indicated by black triangles) in preparations of VLPs (indicated by white arrow) (d), purified PPRV VLPs (e), and immunogold-labelled PPRV VLPs stained with mouse anti-PPRV H polyclonal antibody followed by gold-labeled goat anti-mouse IgG antibody (f). (**g**) Western blot depicting VLP protein expression using mouse anti-PPRV M or H polyclonal antibodies or sheep polyclonal antibody sera against N or F.

The synthetic genes M, F, H and N were each sub-cloned into the Puc57-Simple plasmid (Life Technologies, San Diego, CA, USA) to produce Puc57-Simple-M, Puc57-Simple-F, Puc57-Simple-H, and Puc57-Simple-N, respectively. All the primer sequences are shown in Table 1. Each gene was subsequently cloned into the pFastBacDual vector under a p10 promoter and a pH promoter to produce pFBD-2M, pFBD-2F, pFBD-2H, and pFBD-2N (Figure 1A). Recombinant plasmids were transformed into *Ecoli* DH10Bac (Life Technologies, USA) competent cells, after which recombinant bacmids were identified by PCR using the two primer pairs M13F/P10R and M13R/P10F and purified as previously described [28].

### 2.3. Generation of Recombinant Baculoviruses

Recombinant baculoviruses (rBVs), named rpFBD-2M, rpFBD-2F, rpFBD-2H and rpFBD-2N expressing M, F, H and N proteins, respectively, were rescued using the Cellfection^®^II reagent (Life Technologies, USA) following the manufacturer’s protocol. In brief, 3 μg of bacmid DNA was diluted in 100 μL unsupplemented Grace’s Insect Medium (Sigma, USA) and vortexed briefly. Diluted DNA (100 μL) and 100 μL diluted Cellfectin^®^II reagent were combined and vortexed briefly, followed by incubation at room temperature for 15–30 minutes. DNA–lipid mixture was added onto the Sf9 cells and the cells were incubated at 27 °C for 3 h. Insect medium containing 10% FBS with supplements was used to replace the old medium. The first generation of virus (P1) was harvested after 4 days incubation at 27 °C. In order to obtain high-titer baculovirus, cells were continuously passaged for several generations.

### 2.4. Immunofluorescence Assay (IFA)

Sf9 cells were infected with four rBVs at a multiplicity of infection (MOI) of 1. After 48 hours, Sf9 cells were fixed in 80% acetone for 30 min at room temperature, after which, they were incubated with one of the following primary antibodies: anti-M (1:100 dilution), anti-F (1:1000 dilution), anti-H (1:100 dilution), or anti-N (1:1000 dilution). A FITC-labeled goat anti-mouse antibody (1:200 dilution Millipore, USA) was used as a secondary antibody to detect the expression of PPRV M and F proteins, and a FITC-labeled mouse anti-goat (1:1000 Millipore, USA) was used as a secondary antibody to detect the expression of PPRV H and N proteins. Cells were incubated with primary and then secondary antibodies at room temperature for 30 min each and rinsed three times with PBS (phosphate buffer saline) between steps. The plate was observed using a ZEISS Axio Vert.A1 Inverted Microscope and FITC filter (Zeiss, Germany, Germany). Uninfected Sf9 cells and cells infected with wild-type baculovirus were used as negative and positive controls, respectively. 

### 2.5. Construction and Purification of Virus-Like Particles

To generate PPRV VLPs, Sf9 cells were infected with rBVs at an MOI of 5 in a volume of 300 mL with a cell density of 1–2×10^7^/mL. Cells were cultured in suspension at 27 °C and 120 rpm. At 120 hours post-infection, supernatant was harvested and centrifuged at 5000 rpm for 20 min to remove cells and debris, after which, clarified supernatant was centrifuged at 30,000 rpm for 90 min at 4 °C to pellet the VLPs. VLPs were subsequently purified through a 20%–40%–60% (*w*/*v*) discontinuous sucrose gradient prepared in PBS, with centrifugation at 35,000 rpm for 90 min at 4 °C. Bands between 40% and 60% sucrose, corresponding to purified VLPs, were harvested, and the VLP concentration was determined to be 8–10 mg/L by BCA kit (BCA protein assay kit, China).

### 2.6. Detection of Protein by Western Blot Analyses

Samples collected from Sf9 cells infected with rBVs were treated with 5×SDS Loading Buffer for 10 min at 100 °C and separated using 12% SDS-PAGE gels. For Western blot analysis, samples separated through SDS-PAGE were transferred onto polyvinylidene fluoride (PVDF) membranes (Immobilin-p, Millipore, USA), after which, the membranes were blocked for 1 hour in 5% milk in Tris-buffered saline with Tween 20 (TBST). Two different antibodies were used to detect the presence of M, F, H and N: mouse polyclonal antibody for M protein and sheep polyclonal antibody for N, F, and H protein. HRP-labeled goat anti-mouse IgG (1:10000 dilution; Abcam, USA) and HRP-labeled rabbit anti-sheep IgG (1:10000 dilution; Bioworld) were used as secondary antibodies. The membranes were washed in TBST and visualized using Western Blue stabilized substrate (Promega, USA) to detect alkaline phosphatase.

### 2.7. Electron and Immuno-Electron Microscopy

A copper–rhodium (Cu–Rh) grid with a mesh size of 200 was incubated with VLPs at room temperature for 60 min and then incubated with a 200-fold dilution of sheep anti-PPRV sera produced in-house at 37 °C for 60 min. The copper mesh was then incubated with a 50-fold dilution of 10 nm gold-conjugated goat anti-mouse IgG (Abcam, USA) at room temperature for 60 min. VLPs were stained with 1% phosphotungstic acid, and the excess dye solution was removed after 3 min. Samples were observed under a transmission electron microscope.

### 2.8. Immunization of Mice and Goats and Sera Collection

All live animal work was performed in accordance with guidelines from the Animal Welfare and Ethics Committee of the Changchun Veterinary Research Institute (Permit No. SCXK-2012-017). The environment and housing facilities satisfied the National Standards of Laboratory Animal Requirements (GB 14925-2001) of China.

BALB/c mice (female, 6–8 weeks old) were purchased from Changchun Animal Breeding Center for Medical Research (Changchun, China). Animals were randomized into three groups of 10 and immunized either subcutaneously (s.c.) with 50 μg VLPs in 50 μL PBS mixed with an equal volume of Freund’s complete adjuvant (Thermo Fisher, USA), or immunized s.c. with 10^3^ TCID_50_ PPRV Nigeria 75/1 diluted in 100 μL DMEM. Mice in the control group were given 100 μL of PBS alone. All groups received a second and third immunization two and four weeks following the primary immunization. The booster immunizations were identical to the original immunization, with the exception of the VLP group, in which Freund’s incomplete adjuvant was used instead of Freund’s complete adjuvant. Blood samples were collected by retro-orbital plexus puncture at 2, 4, and 6 weeks post-vaccination. Serum was separated and stored at −80 °C for analysis of antibody and cytokine levels.

Outbred goats (male and female, 6–18 months old) were purchased from the Changchun Institute of Biological Products Co., Ltd, China and randomized into five groups of five animals each. The first and second groups were vaccinated s.c. in the gastrocnemius muscle with 150 or 300 μg/goat of PPRV VLPs, respectively, mixed with an equivalent volume of Imject^TM^ Alum adjuvant (Thermo Fisher, USA). The third group was inoculated with 10^5^ TCID_50_ PPRV Nigeria 75/1 in 1 mL DMEM as a positive control. The fourth group was inoculated subcutaneously with 1 mL PBS or alum adjuvant as a negative control. All goats received a second and third identical vaccination 3 and 6 weeks after the first. Blood was collected through the jugular vein at different time-points post-immunization using 10 mL vacuum blood collection tubes; serum was separated and stored at −80 °C for antibody titration.

### 2.9. ELISA for Antibody Subtype Detection

Mouse sera obtained at 2 weeks post-immunization was analyzed for PPRV-specific IgM, while sera obtained at 6 weeks post-immunization was analyzed for PPRV-specific IgA, IgG, IgG1, and IgG2a. Antibody titers were measured using an indirect ELISA, as described elsewhere [26]. The coating concentration of purified PPRV whole-virus antigen was 2 mg/mL, and the optical density (OD) value was recorded at 450 nm absorbance.

### 2.10. Virus Neutralization Test (VNT)

PPRV-specific virus neutralization antibody (VNA) titers were analyzed in serum samples collected from immunized mice and goats using a micro-neutralization assay. Serially diluted (mouse sera, from 1:4; goat sera, from 1:5) inactivated serum was mixed with an equal volume of PPRV (10^3^ TCID_50_/mL) in a 96-well cell culture plate. Virus-only control wells and uninfected-cell control wells were also included. After incubation at 37 °C for 1 h, Vero cell suspension was added (50 mL/well). The cytopathic effect (CPE) was observed after 8 days incubation. VNA titer was expressed as the highest dilution at which the CPE was inhibited by at least 50%, according to previously described methods [26].

### 2.11. IFN-γ and IL-4 Enzyme-Linked Immunospot Assays (ELISpot)

Two weeks after the second immunization, splenocytes were obtained from mice and stimulated with inactivated PPRV (10 µg/mL). IFN-γ- or IL-4-secreting cells were quantified using ELISpot kits (Mabtech AB, Stockholm, Sweden; R&D Systems, Minneapolis, MN, USA), according to the manufacturer’s instructions. Spot-forming cells (SFCs) were counted on an automated ELISpot reader (AID ELISPOT reader-iSpot, AID GmbH, GER) [29,30].

### 2.12. Flow Cytometry Assays for Intracellular Cytokine Staining (ICS)

Mouse splenocytes (1×10^7^ cells/mL) were harvested two weeks after the second vaccination. Cells were stimulated with inactivated PPRV (10 μg/mL) containing monensin (BD Biosciences, Franklin, TN, USA) for inhibiting protein transport. At 6 h post-stimulation, anti-CD8 and anti-CD4 monoclonal antibodies (BD Biosciences) were used to quantify surface expression of CD8 and CD4. After permeabilization with Cytofix/Cytoperm (BD Biosciences), cells were stained with anti-IFN-γ and anti-IL-4 monoclonal antibodies (BD Biosciences). All stained cells were detected using a FACSAria TM CELL Sorter (BD Biosciences), as previously described [29,30].

### 2.13. Flow Cytometry Assays for B Cells and DCs

Activation of B cells and DCs was analyzed using a flow cytometry assay, as previously described [29,30]. Briefly, inguinal lymph node samples were collected from mice two weeks after the second immunization. The cell suspensions were labelled with primary monoclonal antibodies against cell-surface molecules CD19, CD40, CD11c, CD80, CD86, MHC I, and MHC II (BD Pharmingen). All stained cells were detected using FACSAria TM CELL (BD Biosciences).

### 2.14. ELISA for PPRV-Specific Antibody and Cytokine in Goat Sera

To determine antibody responses to the F, H, and N protein of PPRV, an indirect ELISA was developed using purified F, H, and N proteins. The three soluble proteins were produced in BL21 (DE3) bacteria (Takara) transformed with a T7 promotor plasmid that expresses the open reading frame of F, H, or N protein fused to a His-tag. Purification of the His-tagged protein from the inclusion bodies was done using HisPurTM Ni-NTA Spin Columns (Thermo Fisher, USA), as per the manufacturer’s protocol. Purified F, H, and N proteins (0.5 µg) were used to coat 96-well, flat-bottomed plates overnight at 4 °C. The following steps were performed according to a previous study [26]. The total IgG and IgM were detected using a similar method: 96-well plates were coated with 0.05 µg purified inactivated PPRV Nigeria 75/1, and HRP-conjugated rabbit anti-goat IgG (1:10,000 in PBST; ThermoFisher) and IgM (1:10,000 in PBST; ThermoFIsher) were used as secondary antibodies.

To determine cytokine responses, Goat Interleukin 4 (IL-4), Goat Interleukin 10 (IL-10) and Goat Interferon γ (IFN-γ) ELISA Kits were purchased from Cusabio. The reagent, samples and standards were prepared according to the manufacturer’s protocol.

### 2.15. Data Analysis

Figures were generated using GraphPad Prism 8.0 software. Differences between means were evaluated using the one-way ANOVA or two-way ANOVA and were deemed significant at P values of 0.05 or less.

## 3. Results

### 3.1. Generation and Characterization of PPRV VLPs

To generate PPRV VLPs, we first constructed a series of bacmids, each expressing two copies of the codon-optimized genes for M, H, F, or N from two separate promoters (p10 and pH) in order to enhance protein expression (Figure 1a). From these bacmids, four recombinant baculoviruses (rBVs), each expressing a single PPRV structural protein, were rescued, and expression of M, H, F, and N was verified by immunofluorescence (Figure 1b). The four rBVs were then used to simultaneously co-infect Sf9 cells, from which VLPs composed of all four PPRV structural proteins were purified at high concentrations (ranging from 8–10 mg/L). Transmission electron microscopy results showed the presence of baculovirus and PPRV-like particles in the supernatant before purification (Figure 1c). The diameter of purified PPRV VLPs (Figure 1d) was approximately 100–200 nm and was consistent with the size of native PPRV particles (Figure 1e). Immuno-electron microscopy suggested that the PPRV major surface glycoproteins were successfully incorporated into the VLPs (Figure 1f), and incorporation of all four PPRV proteins was confirmed by Western blotting (Figure 1g).

### 3.2. Characterization of the Humoral Immune Response to PPRV VLPs in Mice

**Figure 2:**
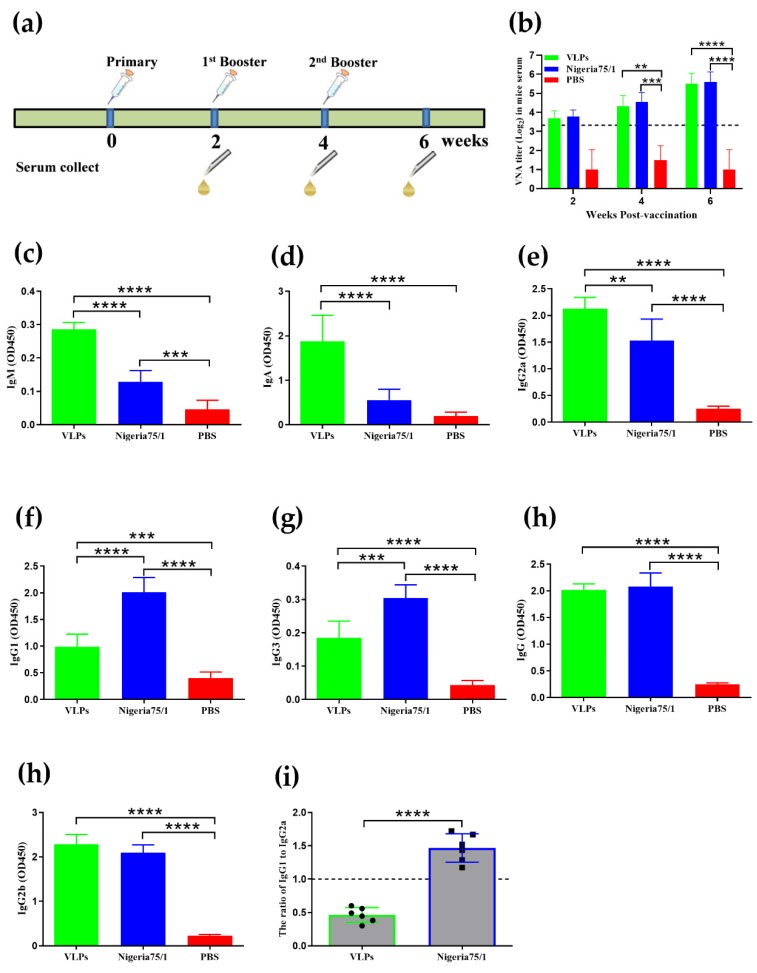
Immunization induces a humoral immune response and Th1 class immunity in mice. (**a**) Mice were vaccinated three times via the subcutaneous route at 2-week intervals with VLPs, PPRV Nigeria 75/1 or PBS. Blood sampling and serum isolation were performed 2, 4, and 6 weeks after the first immunization. (**b**) Virus-neutralizing antibody (VNA) titers were measured by fluorescent antibody viral neutralization (FAVN). (**c–i**) Serum was diluted by a factor of 5000, and total serum IgG and antibody subtypes were quantified by ELISA. The IgM (c) response were evaluated 1 week after the first vaccination, whereas IgA (d), IgG2a (e), IgG1 (f), IgG3 (g), IgG (h), and IgG2b (i) responses were determined 2 weeks after the second immunization. (**j**) The IgG1/IgG2a ratio was measured. Dotted lines represent antibody titers greater than ten, indicating positive serum conversion. Significant differences were analyzed by one-way or two-way ANOVA and indicated as follows: * *P* < 0.05, ** *P* < 0.01, *** *P* < 0.001, **** *P* < 0.0001.

To assess the immunogenicity of VLPs, mice were vaccinated with 50 µg VLP and boosted two weeks later, after which, neutralizing antibody titers were determined using a micro-neutralization assay. Mice immunized with 10^3^ TCID_50_ of the commercial live-attenuated vaccine PPRV Nigeria 75/1 were used as positive controls, while mice immunized with PBS served as negative controls. Inactive sera collected at different time-points after immunization were serially diluted and incubated with live PPRV Nigeria 75/1. The virus was then added to Vero cells, and the development of CPE was monitored. As shown in Figure 2b, two weeks after the initial immunization, the titers of virus-neutralizing antibodies (VNA) in the VLP- and PPRV Nigeria 75/1-vaccinated groups exceeded 10, the standard minimum value required for protection in goats immunized with live-attenuated PPRV vaccine [31]. Both VLP-vaccinated and Nigeria 75/1-vaccinated mice developed neutralizing antibodies, with titers ranging from 1:16 to 1:32 after the second immunization (week 4) and from 1:16 to 1:64 after the third immunization (week 6).

To further investigate the humoral response to PPRV VLPs, antigen-specific total IgG and antibody subtypes were confirmed by ELISA (Figure 2). The total serum IgA and IgM antibody titers from VLP-immunized mice were significantly higher than those observed for PPRV Nigeria 75/1 and the PBS control (Figure 2c,d). Likewise, PPRV VLPs induced a stronger IgG2a response than PPRV Nigeria 75/1 but a weaker IgG1 and IgG3 response (Figure 2e–g). There was no significant difference in total serum IgG and IgG2b among VLP-immunized and PPRV Nigeria 75/1-immunized groups (Figure 2h,i). Interestingly, the low ratio of IgG1 to IgG2a in VLP-immunized mice suggested the activation of Th1-type immune response, whereas the opposite phenomenon in PPRV Nigeria 75/1-immunized mice suggested the activation of Th2-type immunity [29] (Figure 2j). Overall, the data above support the conclusion that VLPs elicit a strong and broad antibody subclass response in mice, similar to that elicited by the Nigeria 75/1 vaccine.

### 3.3. Characterization of the Cellular Immune Response to PPRV VLPs in Mice

**Figure 3:**
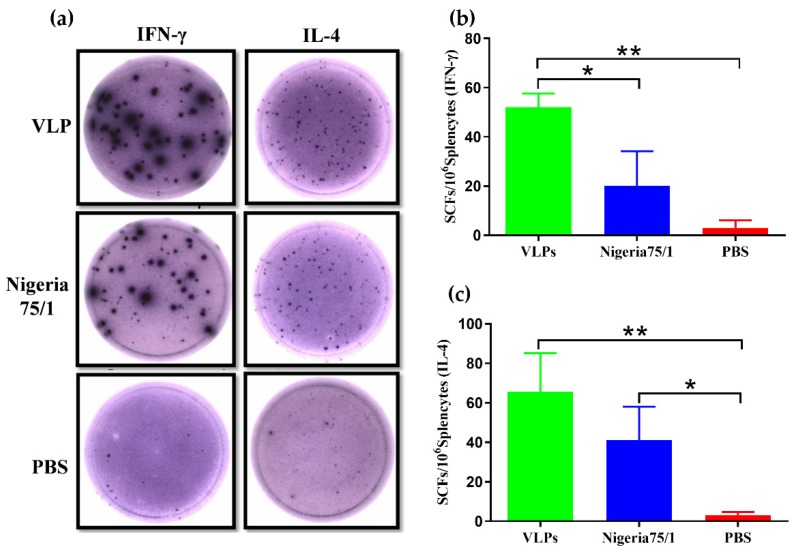
Immunization induces a cell-mediated immune response in mice. Splenocytes from mice were stimulated with inactivated PPRV two weeks after the second immunization. (**a**) Representative images of IFN-γ- and IL-4-secreting splenocytes. (**b,c**) ELISpot assay was used to enumerate IFN-γ-secreting (b) or IL-4-secreting (c) splenocytes. Data are depicted as the mean ± SD spot-forming cells (SFCs) per million splenocytes from three mice in each group and were analyzed by one-way ANOVA (* *P* < 0.05, ** *P* < 0.01,*** *P* < 0.001,**** *P* < 0.0001).

After confirming that VLPs successfully induced a broad antibody response in mice, we next sought to evaluate the induction of a cell-mediated immune response. The antigen-specific IFN-γ and IL-4 activities in splenocytes were evaluated by ELISpot assays (Figure 3a). Spot-forming cells (SFCs) of IFN-γ and IL-4 from the splenocytes of VLP-immunized mice were significantly more numerous than those observed from PBS-immunized mice (Figure 3b,c). Although VLPs were found to induce a much greater IFN-γ response in mice than PPRV Nigeria 75/1, no significant difference in IL-4 responses among VLPs and PPRV Nigeria 75/1 groups were observed.

**Figure 4:**
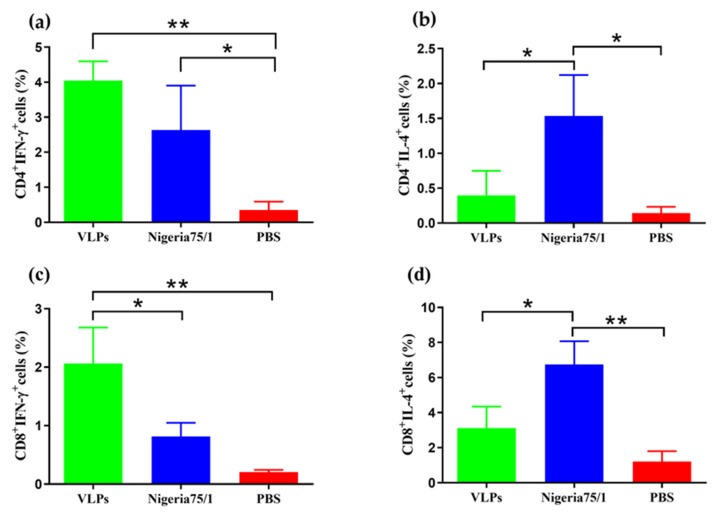
Immunization improves IFN-γ-secreting CD4+ and CD8+ T cell responses in mice. Two weeks after the second immunization, splenocytes from three mice in each group were cultured and stimulated with inactivated PPRV Nigeria 75/1. Mouse monoclonal antibodies against CD4, CD8, IFN-γ, and IL-4 were used to identify cells that were double-positive (**a**) CD4^+^ IFN-γ^+^, (**b**) CD4^+^ IL-4^+^, (**c**) CD8^+^ IFN-γ^+^, and (**d**) CD8^+^ IL-4^+^. Data were analyzed by one-way ANOVA and indicated as follows: * *P* < 0.05, ** *P* < 0.01, *** *P* < 0.001, **** *P* < 0.0001.

To further characterize the ability of PPRV and VLPs to produce specific T cell responses, CD4^+^ and CD8^+^ splenocytes were labeled for IFN-γ or IL-4. VLPs elicited a robust IFN-γ-secreting CD4^+^ T cell response in contrast to PPRV Nigeria 75/1, which elicited a significantly greater IL-4-secreting T cell response (Figure 4a,b). Similarly, the percentage of CD8^+^IFN-γ^+^ T cells was significantly higher in the VLP group compared to the PPRV Nigeria 75/1 group, while the opposite was true for CD8^+^IL-4^+^ (Figure 4c,d). These data demonstrate that mice immunized with VLPs elicited a notably enhanced T cell response induced by IFN-γ.

**Figure 5:**
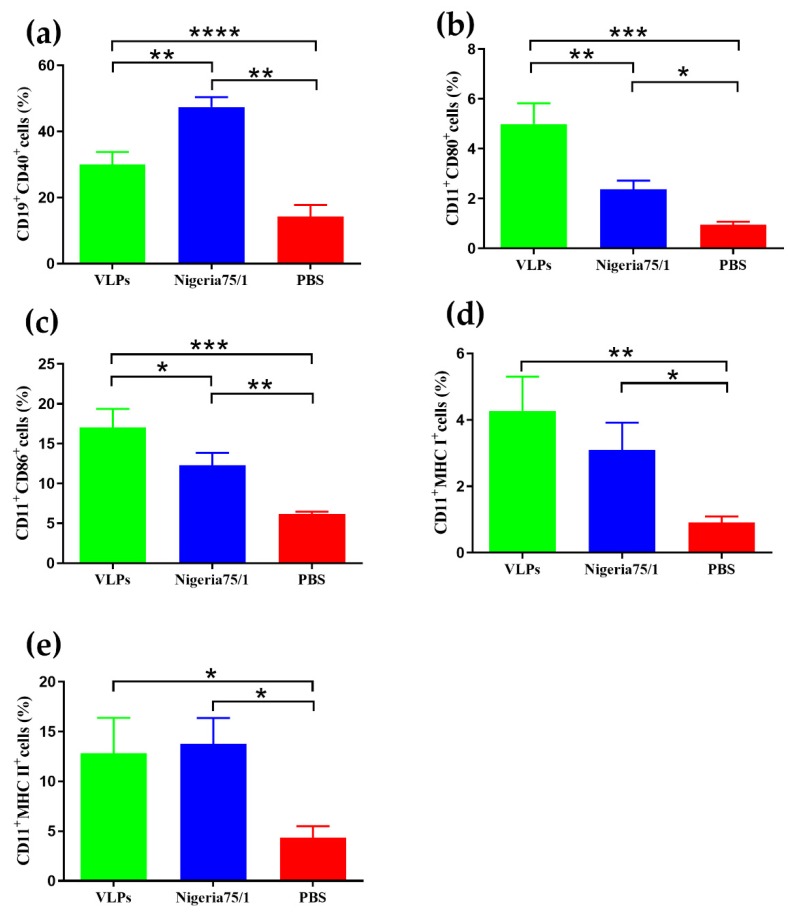
Immunization induces enhanced B cell and dendritic cell recruitment and activation. Two weeks after the second immunization, five mice from each group were euthanized for collection of cells from the inguinal lymph nodes. Mouse monoclonal antibodies against CD19, CD40, CD80, CD86, MCH I, and MCH II were used to identify cells that were double-positive (**a**) CD19^+^CD40^+^, (**b**) CD11c^+^CD86^+^, (**c**) CD11c^+^CD80^+^, (**d**) CD11c^+^MHC I^+^, and (**e**) CD11c^+^MHC II^+^. Significant differences were analyzed by one-way ANOVA and indicated as follows: * *P* <0.05, ** *P* < 0.01, *** *P* < 0.001, **** *P* < 0.0001.

To investigate whether VLPs can induce the production of B cells and dendritic cells (DC), cells from inguinal lymph nodes were analyzed using flow cytometry. Mice immunized with VLPs or PPRV Nigeria 75/1 induced a greater production of CD19^+^CD40^+^ cells (B cells) than PBS alone; however, PPRV Nigeria 75/1 induced significantly higher levels of CD19^+^CD40^+^ cells than VLPs (Figure 5a). Conversely, the levels of CD11c^+^CD80^+^ and CD11c^+^CD86^+^ cells (DCs) in the lymph nodes of VLP-immunized animals were significantly higher than those immunized with PPRV Nigeria 75/1 and the PBS control group (Figure 5b,c). Compared with PBS, both VLPs and PPRV Nigeria 75/1 induced higher levels of MHC I and MHC II expression in vaccinated mice, although there was no significant difference in expression between the VLP- and PPRV Nigeria 75/1-immunized groups (Figure 5d,e). Taken together, these results indicate that VLPs and PPRV Nigeria 75/1 can recruit and/or activate more B cells and DCs in inguinal lymph nodes and also elicit high levels of expression of MHC I and MHC II on the surface of activated DCs.

### 3.4. Characterization of the Humoral Immune Response to PPRV VLPs in Goats

**Figure 6:**
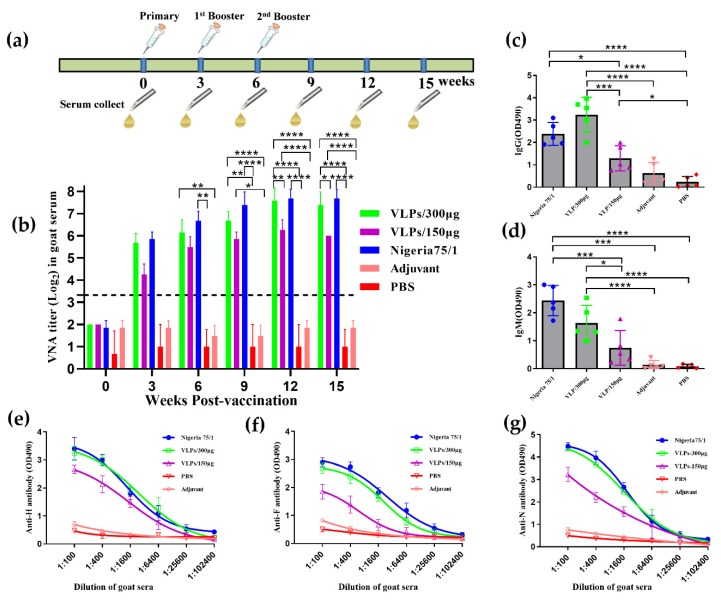
Immunization induces a humoral response in goats. (**a**) Goats were boosted twice every three weeks via the subcutaneous route with VLPs (150 µg or 300 µg), PPRV Nigeria 75/1, PBS, or alum adjuvant after initial immunization. (**b**) Serum samples were collected 3, 6, 9, 12 and 15 weeks after the first vaccination and virus-neutralizing antibody (VNA) titers were measured by fluorescent antibody viral neutralization (FAVN). Dotted lines represent antibody titers greater than ten, indicating positive serum conversion. (**c–d**) Total serum IgG (c) and IgM (d) responses were determined at 3 weeks. (**e–g**) Serum was collected from each goat three weeks after the third immunization for analysis by a PPRV F-, H-, and N-specific ELISA. Significant differences were analyzed by one-way or two-way ANOVA and indicated as follows: *P <0.05, **P <0.01, ***P <0.001, ****P<0.0001.

To evaluate the immunogenicity of VLPs, neutralizing antibody titers in goat serum were determined using a micro-neutralization assay. Goats were immunized with 150 or 300 µg VLPs or 10^5^ TCID_50_ Nigeria 75/1, while control animals were immunized with either PBS or the alum adjuvant alone (Figure 6a). Three weeks after the first immunization, the VNA titers in goats vaccinated with VLPs or PPRV exceeded 10 [31,32] (Figure 6b). Nine weeks after the primary vaccination, the value was significantly increased again, reaching levels of 2^7^–2^8^ in the animals immunized with VLP (300 µg) and PPRV Nigeria 75/1. The data showed that the neutralizing antibody response in PPRV Nigeria 75/1 is higher than that induced by VLPs in goats (target animals), although the differences were not statistically significant. Goats immunized with 300 µg of VLPs induced higher levels of neutralizing antibodies—starting from the third week after the first immunization—compared to goats immunized with 150 µg of VLPs, but the differences were not significant until the twelfth week after immunization, and no neutralization activity was detected in pre-immune serum or in serum from animals immunized with either PBS or adjuvant (Figure 6b). The average levels of IgG (Figure 6c) and IgM (Figure 6d) in animals immunized with 300 µg VLPs were as high as those immunized with PPRV Nigeria 75/1, after the third immunization. Animals immunized with 150 µg VLPs also exhibited a specific IgG and IgM response, although it was significantly lower than the responses elicited by PPRV Nigeria 75/1 and the 300 µg VLP dose. Immune responses against the PPRV F, H, and N proteins were evaluated by ELISA (Figure 6e,f,g). All goats in the VLP- and PPRV Nigeria 75/1-immunized groups developed high levels of antibodies against PPRV F, H, and N proteins after the third immunization, whereas none of the goats immunized with PBS or alum adjuvant alone demonstrated an immune response. We also have used the commercial PPRV ELISA kit (IDvet, France) to repeat the ELISA. The results showed that although the OD values are different from the ELISA method established in this study, the trends of the curves are consistent. Taken together, these data show that VLP immunization induced significantly high levels of serum neutralizing antibodies comparable to PPRV Nigeria 75/1. 

### 3.5. Characterization of the Cytokine Response to PPRV VLPs in Goats

**Figure 7:**
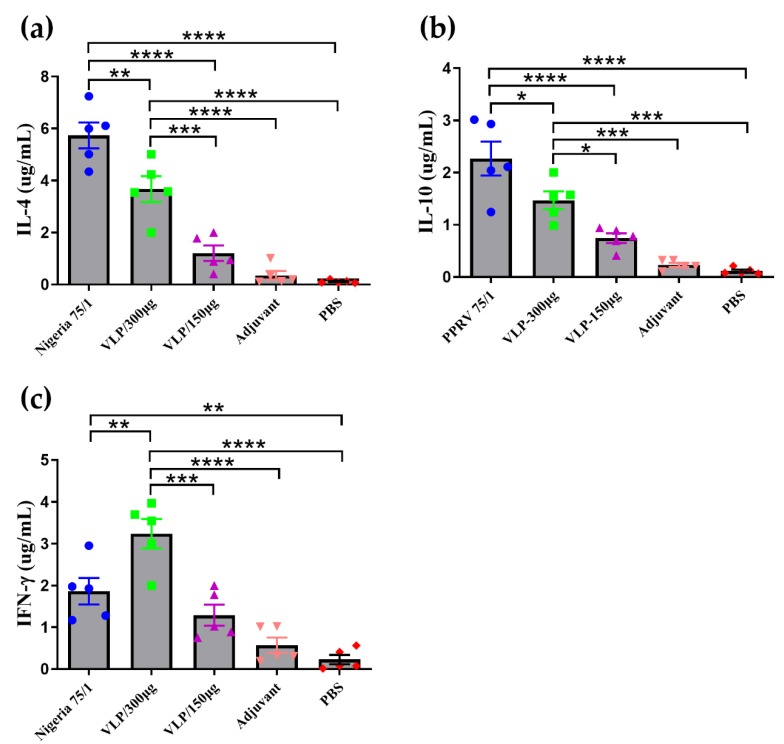
Immunization induces a significant cytokine response in goats. (**a–c**) Serum samples after the second immunization from animals immunized with PPRV VLPs (150 µg or 300 µg), PPRV Nigeria 75/1, PBS, or alum adjuvant were analyzed for IL-4 (a), IL10 (b) and IFN-γ (c). Significant differences were analyzed by one-way ANOVA and indicated as follows: * *P* < 0.05, ** *P* < 0.01, *** *P* < 0.001, **** *P* < 0.0001.

To evaluate the cytokine response induced by PPRV Nigeria 75/1 and VLPs, we quantified the levels of IL-4 (Figure 7a), IL-10 (Figure 7b) and IFN-γ (Figure 7c) in vaccinated animals. Overall, animals vaccinated with 300 µg VLPs exhibited significantly higher levels of these three cytokines than animals given the lower dose or the control animals that received adjuvant or PBS only. Although animals vaccinated with 150 µg VLPs exhibited increased cytokine levels compared to the control animals, the differences were not significant. IL-4 and IL-10 levels induced by PPRV Nigeria 75/1 were significantly higher than in the control animals or animals vaccinated with VLPs, whereas the IFN-γ levels were significantly lower than in animals vaccinated with 300 µg VLPs.

## 4. Discussion

Baculovirus insect cell expression systems are used extensively for the production of VLPs, and they provide several advantages, including fast growth rates in serum-free media, capacity for large-scale cultivation, and ability to create post-translational modifications similar to mammalian cells. In this study, we demonstrate the production of PPRV VLPs at high concentrations using a recombinant baculovirus system to simultaneously co-express the four structural proteins M, F, H, and N.

VLPs that preserve the native antigenic conformation of immunogenic proteins can elicit a strong humoral response by efficiently cross-linking the membrane-associated immunoglobulin molecules constituting the B-cell receptor [33,34,35,36]. This, in turn, enhances the B cell signaling pathway, which stimulates B cell proliferation and migration and has a positive regulatory role for MHC class II molecules, ultimately promoting interaction among B cells and helper T cells to produce a large number of memory B cells [37,38]. Although immunization with PPRV VLPs in combination with Freund’s adjuvant stimulated and recruited more B cells than the PBS treatment, VLPs were less effective at recruiting/activating B cells than PPRV Nigeria 75/1, suggesting that PPRV Nigeria 75/1 may have more B-cell epitopes than PPRV VLPs. Conversely, however, because of the role of adjuvant, VLP-immunized mice may be inclined towards a cellular immune response. Nevertheless, the results presented here show that both VLP- and PPRV Nigeria 75/1-immunized mice recruited significantly high levels of activated B cells in the inguinal lymph nodes, which, in turn, induced a higher and broader serum antibody subclass response.

PPRV VLPs were found to induce a neutralizing antibody response as high as that induced by PPRV Nigeria 75/1 in mice, whereas, in goats, the neutralizing antibody response to Nigeria 75/1 was higher than that induced by VLPs, although the differences were not significant. Indeed, compared to PPRV Nigeria 75/1, VLPs elicited a stronger and more enhanced IgM response in mice, reflecting the potent immunogenicity of the VLPs. In addition, production of IgA was also significantly higher in mice vaccinated with VLPs compared to PPRV Nigeria 75/1, consistent with a previous report that demonstrated increased amounts of IgA production following parvovirus VLPs immunization in mice [39]. As a result, vaccination of animals against PPRV using PPRV VLPs could potentially be successful in preventing the entry of the virus through the mucosal route. IgG1 and IgG3 play important roles in complement fixation and opsonization, as well as in the induction of ADCC (antibody-dependent cell-mediated cytotoxicity) [40], and high serum IgG3 titers have been demonstrated to mediate HIV neutralization in HIV-infected individuals [41]. In mice, both VLPs and Nigeria 75/1 induced significantly high titers of IgG3, suggesting a role for this antibody subtype in quickly and efficiently clearing PPRV infection.

Although both the PPRV VLPs and Nigeria 75/1 induced PPRV-specific humoral responses in mice, VLPs elicited a stronger cellular immune response. The relatively low IgG1/IgG2a ratio indicated that PPRV VLPs induced a Th1 immune response profile that favours a cell-mediated immune response. Compared to PPRV VLPs, PPRV Nigeria 75/1 elicited relatively high levels of IgG1 and resulted in a higher IgG1/IgG2a ratio, suggesting that PPRV triggers a Th2-biased response favouring humoral immunity. Interestingly, these results contradict an earlier study demonstrating higher IgG1 and lower IgG2a serum antibody titers after immunization with PPRV Nigeria 75/1 [26]. Flow cytometry analyses for intracellular cytokine staining showed that VLPs induced a significantly stronger IFN-γ response in CD8^+^T cells compared to PPRV Nigeria 75/1-immunized mice. IFN-γ can significantly enhance the expression of MHC II molecules, promote the activation of helper T lymphocytes, amplify the recognition stage of the immune response, and thus enhance the Th1 immune response of animals [42]. Moreover, enhanced secretion of IFN-γ inhibited the proliferation of Th2 cells [43]. Conversely, mice vaccinated with PPRV Nigeria 75/1 revealed significantly greater amounts of IL-4-secreting CD4^+^ T and CD8^+^T cells. IL-4 produced by Th2 cells drives the maturation of B cells into plasma cells, resulting in antibody production, isotype-switching and affinity maturation [44]. The T cell immune response generated by the N protein is an important contributing factor to the virus-specific memory T cell immune response [45]. Although the N protein is not capable of inducing neutralizing antibodies in the mouse model, it has been confirmed to induce a strong CD8^+^ T cell-mediated immune response [15]. We have shown that PPRV VLPs produced by rBVs can induce both Th1 and Th2 responses in mice. In goats, VLPs were still able to induce neutralizing antibodies that were sufficient to protect goats against PPRV infection. Notably, although the level of neutralizing antibodies was slightly lower in the VLP-immunized goats compared to the PPRV Nigeria 75/1-immunized goats, there was no statistically significant difference. However, the expression and regulatory mechanisms of related cytokines need to be further investigated.

DCs are the most potent antigen-presenting cells (APCs) in the body, and they play a crucial role in promoting the proliferation and differentiation of T cells. A previous study demonstrated that papillomavirus VLPs bind to DCs and stimulate their maturation, including the upregulation of MHC class I and II, CD80, CD86, and CD40 costimulatory molecules and cytokine production [46,47]. Similarly, our results demonstrate that, in mice, immunization with PPRV VLPs recruits and/or activates more DCs (including CD11c^+^ CD86c^+^cells and CD11c^+^ CD80c^+^cells) in the inguinal lymph nodes than immunization with PPRV Nigeria 75/1. Our results also demonstrate that PPRV VLPs stimulate greater expression of MHC I and MHC II molecules on the surface of DCs. Whether VLPs induce immunity through similar mechanisms in goats requires further research, especially considering the differences in mouse and goat immune systems, including differences in Toll-like receptors. High-level expression of cytokines and co-stimulatory molecules likely elicited enhanced cellular immune and humoral responses compared with PBS-immunized mice. Furthermore, VLPs derived from rabbit hemorrhagic disease virus and hepatitis B virus have been found to be efficiently processed by DCs for cross-presentation of an MHC class I-restricted epitope, a process that induced strong protective cytotoxic T lymphocyte responses [48,49]. Our results are consistent with these studies and indicate the high induction of MHC I on DCs. Overall, these results demonstrate PPRV VLPs can activate DCs efficiently in a mouse study. For a goat study, although the ELISpot assay and flow cytometric analysis would help us understand the mechanism by which VLPs induce immune responses in goats, the lack of commercially available antibodies for flow cytometric analysis of goat cells and the appropriate Kit for ELISpot assay have hindered the development of this experiment.

A previous study showed that PPRV VLPs could be readily assembled by the co-expression of M and N proteins in Sf9 cells infected with rBVs; however, these VLPs did not induce neutralizing antibodies in mice or goats [27]. Although another study demonstrated that PPRV VLPs assembled from M and F proteins or M and H proteins can induce neutralizing antibodies in mice and goats, the yield of these VLPs was low, reaching only 3–4.5 mg/L [26]. Using our unique cloning and rescue strategy, which included codon optimization and duplication of the open reading frames, we were able to generate much higher yields of PPRV VLPs. Moreover, to our knowledge, this is the first report of PPRV VLPs generated from four structural proteins via an rBV co-infection strategy, and we have demonstrated that these VLPs are not only capable of inducing robust humoral and cellular immune responses in mice, but are also capable of inducing neutralizing antibodies in goats. Thus, this study represents a significant advancement in the development of PPRV VLPs-based vaccines.

Our results indicate that PPRV VLPs can produce neutralizing antibodies although the titers are a little lower compared to the attenuated vaccine PPRV Nigeria 75/1. PPRV VLPs also induced a higher level of activation of cell-mediated immune responses. Our results demonstrate that the PPRV VLP-vaccine containing all four proteins is highly immunogenic. In conclusion, we demonstrate that PPRV VLPs can be successfully produced and that the resulting vaccine has the ability to induce cellular and humoral immune responses in mice, as well as neutralizing antibodies and enhanced IFN-γ secretion in goats. These findings suggest that PPRV VLPs containing all four viral proteins (M, F, H, and N) may be a promising vaccine candidate to facilitate the control and eradication of PPR. Whether the VLP vaccine induces a long-term humoral response, like the attenuated vaccines, remains to be investigated.

## Figures and Tables

**Table 1:** Sequences of primers designed in our study.

Primer	Sequence (5'-3')	Restriction Enzyme Site
MF	ACAGCTAGCCGGTCCGATGACCGAGATCTACGAT	NheI+RsrII
MR	GCATGCGCGGCCGCTTACAGGATCTTGAACAG	SphI+NotI
FF	ACAGCTAGCCGGTCCGATGACACGGGTCGCAACC	NheI+Rsr II
FR	GCATGCGCGGCCGCCTACAGTGATCTCACGTA	SphI+NotI
HF	ACAGCTAGCCGGTCCGATGTCCGCACAAAGGGAA	NheI+RsrII
HR	GGTACCGCGGCCGCTCAGACTGGATTACATGT	KpnI+NotI
M13F	GTTTTCCCAGTCACGAC	
M13R	CAGGAAACAGCTATGAC	
P10F	GGGGTATCGACAGAGTGC	
P10R	CGGACCTTTAATTCAACCC	

The sequences of restriction enzyme sites are underlined.
